# Efficient Activation of Reconstructed Rat Embryos by Cyclin-Dependent Kinase Inhibitors

**DOI:** 10.1371/journal.pone.0009799

**Published:** 2010-03-19

**Authors:** Robin L. Webb, Kirk A. Findlay, Michael A. Green, Tina L. Beckett, M. Paul Murphy

**Affiliations:** 1 Department of Molecular and Cellular Biochemistry, University of Kentucky, Lexington, Kentucky, United States of America; 2 Sanders-Brown Center on Aging, University of Kentucky, Lexington, Kentucky, United States of America; 3 Department of Neuroscience, Mayo Clinic Jacksonville, Jacksonville, Florida, United States of America; 4 Department of Microbiology, Immunology and Molecular Genetics, University of Kentucky, Lexington, Kentucky, United States of America; National Institutes of Health, United States of America

## Abstract

**Background:**

Over the last decade a number of species, from farm animals to rodents, have been cloned using somatic cell nuclear transfer technology (SCNT). This technique has the potential to revolutionize the way that genetically modified animals are made. In its current state, the process of SCNT is very inefficient (<5% success rate), with several technical and biological hurdles hindering development. Yet, SCNT provides investigators with powerful advantages over other approaches, such as allowing for prescreening for the desired level of transgene expression and eliminating the excess production of undesirable wild-type animals. The rat plays a significant role in biomedical research, but SCNT has been problematic for this species. In this study, we address one aspect of the problem by evaluating methods of activation in artificially constructed rat embryos.

**Principal Findings:**

We demonstrate that treatment with a calcium ionophore (ionomycin) combined with a variety of cyclin-dependent kinase inhibitors is an effective way to activate rat embryos. This is in contrast to methods developed for the mouse embryo, which tolerates much less specific chemical treatments. Methods developed to activate mouse embryos do not translate well to rat embryos.

**Conclusions:**

Activation methods developed for one species will not necessarily translate to another species, even if it is closely related. Further, the parthenogenic response to chemical activators is not always a reliable indicator of how reconstructed embryos will react to the same activation method. A better understanding of rat oocyte physiology, although essential for developing better models of disease, may also provide insights that will be useful for making the SCNT process more efficient.

## Introduction

The modeling of disease processes *in vitro* (*e.g.*, in cultured cells) and through the use of computer simulations is currently far from sufficient to mimic both the systemic effects of new drugs and the complex symptomology of most diseases. Unfortunately, many human diseases have no counterpart in other species. This is a major obstacle to the understanding of disease progression and the development of therapeutics. For these reasons, genetically modified animals expressing one or more disease genes are a vital resource for both the academic and private sectors, and are an indispensible research tool for advancing our understanding of both basic biology and human disease. Currently, the most common genetically modified mammalian model is the mouse. A simple PubMed search of reports of genetically modified animals indicates that ∼97% of the total involve mice.

However, the mouse is not always the ideal option for biomedical research. There are many situations where a genetically modified rat is a preferred model. The first and simplest consideration is size. The rat is larger, making it better suited for microsurgical manipulation, and it provides more tissue for analysis. Second, rats are preferred for research involving behavioral tests [1], an essential consideration for modeling psychiatric and neurological disorders. Rats are also essential for ADMET (Absorption, Distribution, Metabolism, Excretion, and Toxicity) testing, and for basic physiological studies. Finally, for a variety of reasons, modeling some biological processes in mice often fails to adequately mimic the situation in humans. As just one example, the cytokine interferon-γ (IFNγ) exacerbates multiple sclerosis in humans [2] and in the corresponding rat model (experimental autoimmune encephalomyelitis) [3]. In contrast, IFNγ has a protective effect in the mouse experimental autoimmune encephalomyelitis model [4]. Thus, the rat model of multiple sclerosis predicts the human response while the mouse model gives the opposite result.

The vast majority of rodent disease models are generated by the process of pronuclear microinjection (PMI) [5]. This process requires the injection of DNA directly into the pronucleus of a mouse zygote, and then transferring groups of zygotes into pseudopregnant mothers for development. Only 10–20% of the resulting offspring will likely express the transgene; of these, approximately 70% will transmit the transgene through the germ line, making them suitable colony founding animals. The situation becomes more complex when the size of the transgene construct increases. This affects the number of copies that integrate, with a huge range of possibilities: from one to five copies for large transgenes, to several hundred for small transgenes (reviewed in [6]). The investigator has little control over such parameters, and a true assessment of success cannot begin until the animals are born, at which point transgene expression and development of the desired phenotype can be evaluated.

Over the past several years, somatic cell nuclear transfer (SCNT) has proven to be a viable alternative to PMI [7], [8], [9], [10], [11], [12], [13], [14]. This process involves transfer of a somatic cell nucleus into a cytoplast (enucleated oocyte), which must be artificially activated to drive the developmental regime from the single celled oocyte to the whole organism [15]. Using SCNT the investigator specifically selects the genetically modified clonal cell line that will be the source of nuclear material from which to generate a complete animal. Thus, rather than being forced to rely on chance, the investigator has the ability to choose the desired modification or level of expression in advance. This can be critically important if either high levels of expression are necessary to obtain a phenotype, or if the desired model is a targeted alteration in a specific gene (knock-in or knock-out). The ability to screen the cell line for the genetic modification of the investigator's choice gives unparalleled control over the characteristics of the founding animal. This is a major advantage over pronuclear microinjection, where expression of a transgene is highly variable and difficult to control, or in the generation of chimeras from modified embryonic stem (ES) cells.

All animals produced using SCNT will be genetically modified. Nontransgenics are not created in SCNT, since all of the procedural inefficiency occurs during *in vitro* manipulation or through the loss of reconstructed embryos *in utero*. Thus, SCNT results in a reduction in the number of animals that have to be euthanitized, one of the three Rs of animal research (reduction, refinement and replacement), working towards the goal of improving the humane treatment and use of experimental animals.

In spite of the potential advantages of rat models of human disease, few actually exist. Although the second most common transgenic mammal after the mouse, the rat is still highly under utilized, outnumbered by mice almost 50∶1 in the PubMed database. Although PMI was originally developed for mouse embryos, the technology was adapted for generation of genetically modified rats in the 1990s. However, increased space and cost requirements often limit the production of genetically modified rats in this manner [16]. Also, standard SCNT has proven to be very difficult to implement in the rat [17], [18], [19], [20], [21], [22], [23]. To date, only a single report [24] exists describing the successful generation of a rat by this method.

Many factors contribute to these inefficiencies, including inadequate culture conditions for rat embryos (known to spontaneously activate) [25], variations in efficiency using different cell types as donors of either nuclear material and/or cytoplasts [19], [26], inefficient means of activation, and countless others. In this study, we address one of the major obstacles to the successful generation of genetically modified rats by SCNT, the poor efficiency of embryo activation. We report that rat embryos, unlike those of mice, require more selective forms of chemical treatment.

## Materials and Methods

### Animals

Long Evans Hooded (LEH) rats, approximately 2–3 months old, from Harlan Laboratories (Indianapolis, IN) were used as oocyte donors. Mice (B6C3F1, 3–4 weeks old) were used for comparison experiments. Animals were housed in an environmentally controlled room with a 14∶10 hour light∶dark cycle and given food and water *ad libitum*.

### Ethics Statement

All animal work was conducted with prior IACUC approval, and was performed in accordance with USDA and PHS guidelines.

### Oocyte Collection

Female LEH rats were injected intraperitoneally with 40 I.U. pregnant mare serum gonadotropin (PMSG) between 5:00 and 6:30pm and 40 I.U. human chorionic gonadotropin (hCG) approximately 48 hours later. Rats (typically 3–4/experiment) were euthanitized 15–16 hours post hCG by CO_2_ asphyxiation followed by decapitation. We determined in a pilot study that this time point minimizes spontaneous activation in rat oocytes (spontaneous activation rate, determined by abortive division on overnight culture: 15–16 hours, 7/74, 9.5%; 17–18+ hours, 24/112, 21.4%). We were unable to reliably obtain oocytes at 14 hours or less post hCG in the rat. Mice received a lower hormone dose (5 I.U. PMSG/5 I.U. hCG). Oviducts were removed from the females into a prewarmed dish of potassium simplex optimized media (KSOM) with 10 mM HEPES (flushing and handling media, or FHM; Millipore, Danvers, MA). Ampullae were torn with forceps and cumulus cell oocyte complexes moved into a dish containing KSOM and hyaluronidase (1 mg/mL; Sigma-Aldrich, St. Louis, MO), which was then placed into a 5% CO_2_ incubator at 37°C for 5–6 minutes. Remaining cumulus cells were removed by gentle pupating. Denuded oocytes were washed approximately ten times by moving them sequentially through 50 µL drops of KSOM containing 5 µg/mL cytochalasin B (CB; EMD Biosciences, Gibbstown, NJ) [27].

### Oocyte Enucleation and Embryo Reconstruction

**Figure 1:**
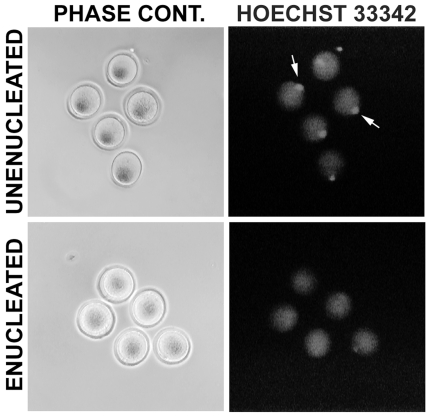
Generating cytoplasts by oocyte enucleation. Prior to insertion of a foreign nucleus, the native genetic material is removed from the oocyte. Enucleation can be performed without the use of fluorescent dyes; oocytes shown were first enucleated, and then briefly stained with Hoechst 33342 to determine efficiency. The same oocytes are shown for both phase contrast and fluorescence microscopy (left and right panels, respectively). The metaphase plate is clearly visible in all 5 examples of unenucleated oocytes (shown by arrows), with no staining evident in any of the enucleated cells.

After collection and washing, oocyte chromosomes were aspirated using a piezo impact micromanipulator (PrimeTech PMM-150FU; Eppendorf, Hauppauge, NY) and a pulled glass micropipette connected to a Cell Tram Vario (Eppendorf). In some cases, enucleation was confirmed by brief exposure to UV light after staining with Hoechst 33342 (Sigma-Aldrich). Accuracy of enucleation was >95% (3 independent experiments: 15/16, 36/36 and 43/44 enucleated successfully; Figure 1). Unenucleated oocytes used in parthenogenic activation experiments were washed to remove hyaluronidase and then placed back into the incubator (at 37°C) for two hours. This incubation was intended to mimic the time required for oocyte enucleation and reconstruction through SCNT.

**Figure 2:**
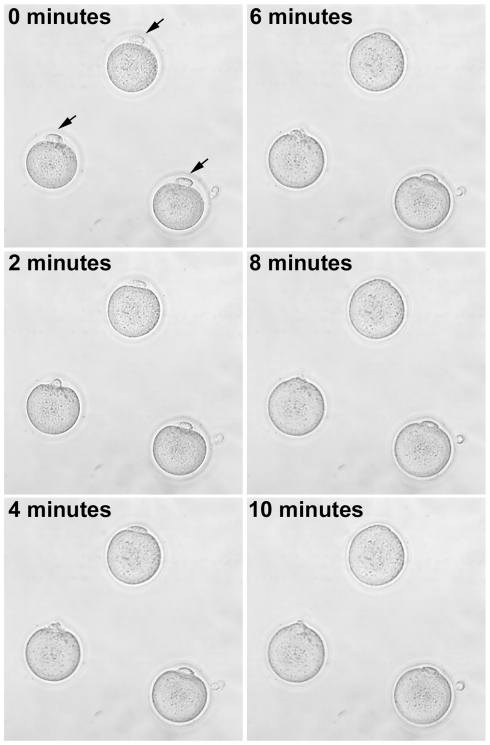
Example of electrofusion of rat fibroblasts with enucleated cytoplasts. A series of bright field photomicrographs are shown over the span of 10 minutes in the fusion chamber after the administration of the electrical pulse (arrows indicate the position of the fibroblasts at time zero). In this case, fusion is essentially complete after 10 minutes.

Embryonic fibroblasts (either rat or mouse, where applicable) were inserted into the perivitelline space with the same pipette used for enucleation. The oocyte-fibroblast complexes that did not spontaneously lyse after 30 minutes at 37°C were sequentially equilibrated by moving them through drops of 25%, 50%, and 75% fusion medium (0.25 M D-sorbitol, 0.1 mM CaOAc, 0.5 mM MaOAc, 0.1% BSA) diluted in KSOM prior to placement in the fusion chamber. Oocyte-fibroblast complexes were arranged such that the fibroblast in the perivitelline space was perpendicular to the electrodes. Fusion was stimulated by an AC alignment pulse (8–10 V AC, 10–15 s), followed by a square wave DC pulse (3.3–3.8 kV/cm DC, 10–15 µs), followed by an additional AC pulse to stabilize membrane contact (8–10 V AC, 5 s). We were also able to achieve fusion in more conductive medias (25 mM NaHCO_3_ or Na_2_HPO_4_, 0.1 mM CaCl_2_, 3.2 mM KCl, 96 mM NaCl; DC pulse of 1.2 kV/cm, 20 µs, with no AC pulse). Fusion was performed with a 1 mm electrode separation using an ECM2001 electro cell manipulator connected to an Enhancer 400 oscilloscope (BTX; Holliston, MA) in order to precisely control conditions (Figure 2). After the electrical pulse, oocyte-fibroblast complexes were left in the fusion chamber for 10 minutes and were then moved to KSOM media. Fusion was assessed following a 30 minute incubation at 37°C. Oocyte-fibroblast complexes that did not fuse were re-equilibrated in the fusion buffer and the electrical stimulation repeated. Reconstructed embryos were washed several times and then placed into drops containing various types of activation media.

### Activation of Collected Oocytes

Following the incubation period, the oocytes were moved sequentially through multiple 50 µL drops of FHM, and then divided into groups. Activation treatments were all conducted in the 37°C incubator. Basic activation conditions consisted of (alone or in combination): 10 mM SrCl_2_ (Sigma-Aldrich) for 3 hours, 5 µM ionomycin (EMD biosciences) for 15 minutes, or 2 mM 6-dimethylaminopurine (DMAP; Sigma-Aldrich) for 2–3 hours. Time of exposure was based on published studies, or in cases where no comparable studies were available, on small scale pilot experiments. Exposure to SrCl_2_ is a commonly used method of activating murine oocytes [28], [29]. Ionomycin treatment is thought to mimic the intracellular calcium spike that occurs immediately in the oocyte following fertilization [30]. Incubation with a calcium ionophore such as ionomycin followed by the general phosphokinase inhibitor DMAP has been used successfully in multiple species, including successful SCNT in both cattle [14] and goats [31]. DMAP is believed to induce activation by preventing the phosphorylation of cdc25, which is normally responsible for activating maturation promoting factor, or MPF [30]. Since DMAP is relatively nonselective, we also tested the activation efficacy of 3 different cyclin dependent kinase inhibitors (CDKIs; 100 µM, 4–6 hours): bohemine [32], roscovitine [33], and butyrolactone [24]. Hence, our range of activation conditions covered a broad spectrum, from the relatively crude addition of strontium to the media, to the relatively specific treatment with CDKIs. Following the activation procedure, embryos were washed at least 10 times and placed in drops of KSOM (for mouse embryos) or mR1ECM (modified rat 1-cell embryo culture medium, for rat embryos) for further development *in vitro* [24], [34]. Activation state was assessed ∼24 hours later. Activation was defined as the number of embryos to undergo at least a first stage cleavage (to 2-cell embryo). Embryos that had undergone an additional cleavage to 4-cell were pooled with the 2-cell embryos to determine the total number activated. Statistical significance was determined using the χ^2^ test, corrected for multiple comparisons by the Holm-Bonferroni method [35].

## Results

We first tested methods known to work reasonably well for the activation of mouse embryos (Table 1). Strontium was an effective means of activation [χ^2^_(1)_ = 5.66, p<0.05], for both parthenogenic mouse embryos and for those that were reconstructed following enucleation and electrofusion. Ionomycin followed by DMAP (I+DMAP) treatment also worked well [χ^2^_(1)_ = 6.60, p<0.05]. Overall effectiveness of activation did not differ between parthenotes and reconstructed embryos in the mouse. However, although we were able to culture mouse parthenotes to the blastocyst stage (38.1%) following both strontium [χ^2^_(1)_ = 6.78, p<0.05] and I+DMAP [χ^2^_(1)_ = 9.45, p<0.05] activation, a much smaller fraction of reconstructed embryos (7.5%) were able to develop to this point [χ^2^_(1)_ = 9.30, p<0.05]. Only reconstructed mouse embryos activated with strontium developed to the blastocyst stage. However, preliminary tests in rat embryos indicated that strontium failed as a means to activate reconstructed rat embryos. This lead us to pursue other methods of activation.

**Table 1:** Mouse Oocyte Activation.

*Parthenogenic:*		
*Treatment*	*Activated*	*Blastocyst*
**None**	18/65 (27.7)	0/18 (0.0)
**Sr^2+^**	23/57 (40.4)	10/23 (43.5) *
**I + DMAP**	22/63 (34.9)	14/22 (63.6) *

*Reconstructed:*		
*Treatment*	*Activated*	*Blastocyst*
**None**	1/18 (5.6)	0/1 (0.0)
**Sr^2+^**	24/42 (57.1) *	4/24 (16.7)
**I + DMAP**	28/37 (75.7) *	0/28 (0.0)

Activation data are numbers of normal appearing 2–4 cell embryos after 24 hours/total (%). Number of blastocysts with a defined inner cell mass were determined following 7 days of culture. Data are compiled from 3 separate experiments (*  =  p<0.05, compared to untreated group).

**Figure 3:**
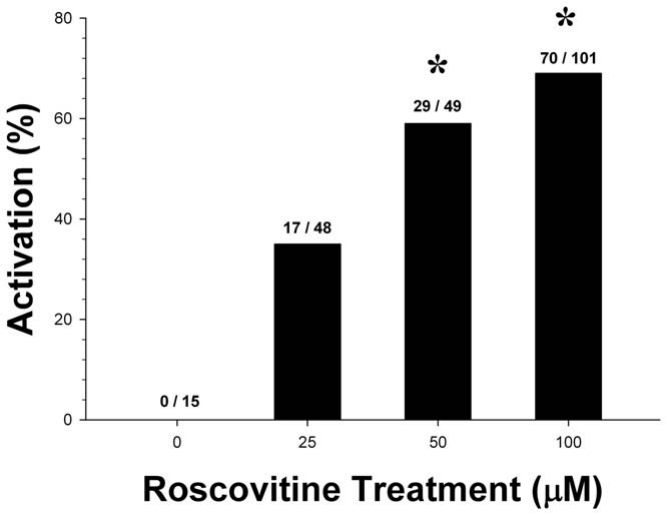
Roscovitine dose response. Roscovitine was able to trigger parthenogenic activation in rat oocytes. The difference between the rate of activation in the 50 µM and 100 µM treatment conditions was not significant. Data shown are compiled from three separate experiments (*  =  p<0.05, compared to control group).

We decided to refine the I+DMAP treatment, which is a moderately more targeted activation treatment than strontium exposure. DMAP is relatively nonspecific, blocking numerous other kinase activities, and can lead to the abnormal mobilization of cytoskeletal components and premature mitosis [36], [37]. We reasoned that we might achieve better results by substituting more specific cyclin-dependent kinase inhibitors (CDKIs) in the place of DMAP. We selected bohemine [32], roscovitine [33], and butyrolactone [24], which are purine analogs that bind within the ATP binding pocket. This mimics the sperm induced down-regulation of cytostatic factor(s) that are responsible for maintaining levels of MPF, leading to the proteolytic degradation of cyclin B and subsequently allows the oocyte to complete the second meiotic division and begin development [30]. Good rates of activation were achievable with roscovitine [χ^2^_(3)_ = 13.1, p<0.05], starting at a concentration of 50 µM [χ^2^_(1)_ = 8.1, p<0.05] to 100 µM [χ^2^_(1)_ = 9.85, p<0.05] (Figure 3), even though roscovitine is typically used at lower concentrations [38], [39]. Similar results were obtained with bohemine (not shown).

**Figure 4:**
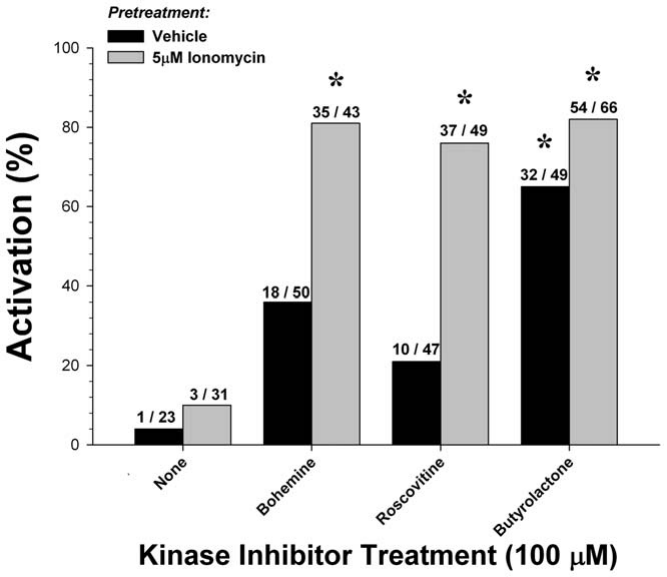
Activation of rat oocytes with cyclin-dependent kinase inhibitors. Approximately half the oocytes recovered from each dissection were incubated with 5 µM calcium ionophore ionomycin for 15 minutes preceding cyclin-dependent kinase inhibitor treatment. Parthenogenic activation using CDKIs with ionomycin pretreatment (68.1%) was more effective than no pretreatment (36.1%). Data shown are compiled from five separate experiments (*  =  p<0.05, compared to control group).

We selected a concentration of 100 µM to compare the three CDKIs, alone or in combination with ionomycin, as a means of activating rat parthenotes (Figure 4). Ionomycin treatment alone failed as a means of activation. CDKI treatment alone was able to activate rat oocytes [36.1%; χ^2^_(3)_ = 15.5, p<0.01]. Although butyrolactone was the most potent activator [65.3%; χ^2^_(1)_ = 10.7, p<0.01], there were no significant differences between any of the three CDKIs. Pretreatment with ionomycin significantly improved activation [68.3%; χ^2^_(3)_ = 11.6, p<0.01]. Bohemine [81.4%; χ^2^_(1)_ = 13.7, p<0.01], roscovitine [75.5%; χ^2^_(1)_ = 12.8, p<0.01] and butyrolactone [81.8%; χ^2^_(1)_ = 14.9, p<0.01] were all equally potent.

**Figure 5:**
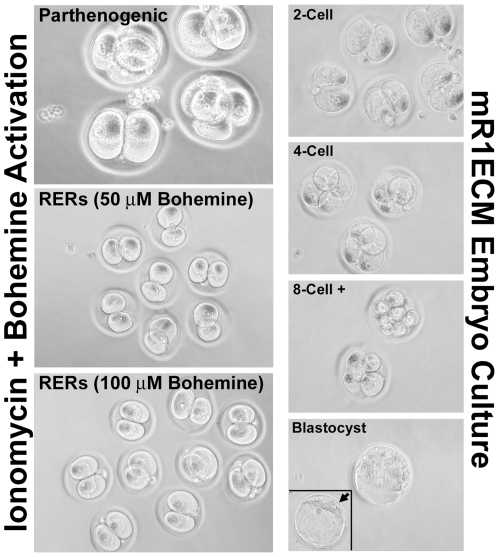
Culture and development of rat embryos. Left panels illustrate parthenogenic and reconstructed embryo activation using the calcium ionophore ionomycin followed by bohemine treatment. There were no apparent differences between oocytes treated with 50 µM versus 100 µM bohemine, and activated parthenotes were indistinguishable from activated reconstructed rat embryos (RERs). Right panels illustrate the development of rat embryos in mR1ECM medium. Less than 5% of rat embryos develop beyond the 4-cell stage, with only ∼1% developing to the blastocyst stage (*bottom panel, insert*: arrow indicates blastocyst inner cell mass).

We next tested the hypothesis that the combination of ionomycin pretreatment followed by exposure to a CDKI would be an effective means of activating reconstructed rat embryos (Table 2). Since the three CDKIs were all very similar in our preliminary tests, we elected to examine bohemine in more detail, as a representative example for a side by side comparison. Parthenotes activated far more readily than reconstructed embryos [χ^2^_(1)_ = 26.2, p<0.01], by a more than 2∶1 ratio (175/493, 35.5% *vs.* 68/424, 16.0%). In contrast, reconstructed embryos were more adversely affected by activation treatments [χ^2^_(1)_ = 23.5, p<0.01] than parthenotes by nearly the same margin (83/493, 16.8% *vs.* 148/424, 34.9%). This appeared to be a general phenomenon in the reconstructed embryos. Although both strontium [χ^2^_(1)_ = 37.1, p<0.01] and I+DMAP [χ^2^_(1)_ = 31.6, p<0.01] were very effective at activating parthenotes, neither were effective for reconstructed embryos. However, bohemine combined with ionomycin was equally effective for both parthenotes [51.3%; χ^2^_(1)_ = 34.2, p<0.01] and reconstructed embryos [53.5%; χ^2^_(1)_ = 47.9, p<0.01]. After activation, and in a follow-up study, we continued to culture rat embryos for an additional 5–7 days, to determine the rate of blastocyst formation (Figure 5). Even in mR1ECM media, widely considered the best for rat embryo culture, we estimate that <2% of embryos are able to develop to this stage under these culture conditions. We confirmed these observations by culturing normal, fertilized rat oocytes collected from normally mated rats in our animal colony. In data pooled from two separate experiments using different batches of mR1ECM, only 2.6% progressed beyond the 4-cell embryo stage (8/302), and only 1.3% (4/102) progressed to blastocysts.

**Table 2:** Rat Oocyte Activation.

*Parthenogenic:*		
*Treatment*	*Activated*	*Degenerate*
**None**	14/156 (9.0)	15/156 (9.6)
**Sr^2+^**	23/29 (79.3) **	6/29 (20.7)
**Ionomycin (I)**	16/62 (25.8)	7/62 (11.3)
**I + DMAP**	61/127 (48.0) **	21/127 (16.5)
**I + Bohemine**	61/119 (51.3) **	34/119 (28.6) *

*Reconstructed:*		
*Treatment*	*Activated*	*Degenerate*
**None**	11/188 (5.9)	62/188 (33.0)
**Sr^2+^**	6/70 (7.4)	31/70 (44.3)
**Ionomycin (I)**	2/30 (3.3)	3/30 (10.0)
**I + DMAP**	3/50 (6.0)	35/50 (70.0)
**I + Bohemine**	46/86 (53.5) **	17/86 (19.8)

Activation data are the number of normal appearing 2–4 cell embryos after 24 hours/total (%). Degenerate embryos are those that exhibited extensive fragmentation/uneven division of the ooplasm. Data are compiled from a minimum of 3 separate experiments (*  =  p<0.05, ** =  p<0.01, compared to untreated group).

## Discussion

Although there is a large, unmet need for genetically modified rats (transgenic, knock-in and knock-out), such animals are rare. The reason for this is simple: the various techniques used to make genetically modified mice do not work as well in rats, are extremely inefficient, or have proven technically unfeasible. Rat embryonic stem (ES) cells are not widely available for gene targeting approaches, and only recently has an ES cell-free method for making knock-out rats been reported [40]. For creating transgenic animals, pronuclear microinjection (PMI) is far less efficient in rats as compared to mice, and most animal facilities are not equipped to accommodate the large rat colonies necessary for this trial and error approach. Other alternatives to PMI (*e.g.*, lentiviral vectors) are unable to generate high expressing lines that can be maintained over multiple generations [41], and are of limited use. Thus in spite of a need for genetically modified rats as an important alternative to mice, developing such models has simply been beyond the reach of most investigators. The recent development of nuclear transfer techniques to produce animals from somatic cells offers a potential alternative to the traditional approach to transgenesis. SCNT, or “cloning”, has so far been applied successfully to sheep [15], [42], cattle [14], [43], goats [31], [44], pigs [45], [46], cats [47], rabbits [48] and mice [28], among others. The technique has also been used to create genetically modified animals [14], [31], [44], [49], [50]. However, adapting SCNT for use in the rat has proven very difficult. To date, only a single report [24] exists describing the successful generation of a rat by this method. In this study, we report the use of cyclin-dependent kinase inhibitors coupled with calcium ionophore treatment to achieve the efficient activation of reconstructed rat embryos, a finding that will improve the probability of eventually discovering the correct combination of conditions for successful rat SCNT.

It is interesting to note that strontium chloride exposure appears to be an excellent method of activation for mouse reconstructed embryos [28], [51], [52], but a poor method for rat reconstructed embryos. Hence, activation methodology will not necessarily translate between species, even if they are closely related. Treatment with strontium chloride was effective as an activator for both mouse parthenotes and reconstructed embryos, and reconstructed mouse embryos were able to develop to the blastocyst stage using this treatment. This is in marked contrast to the results obtained in rat embryos: activation levels induced by strontium chloride in rat parthenotes were comparable to that of the mouse, but yet this treatment could not activate reconstructed rat embryos. These results are very similar to those of Hayes *et al* [20], who also had no success with I+DMAP. This group was most successful at activating rat embryos with an ethanol/cycloheximide treatment protocol, although no live births were obtained. Cycloheximide is a nonspecific inhibitor of protein synthesis, with activation being induced indirectly through the inhibition of cyclin B production [30], [53]. Cycloheximide also depletes the oocyte of proteins required for DNA synthesis, resulting in abnormal DNA content and a significant delay in development [54]. Even though ethanol/cycloheximide has been used to clone cattle [55], post-implantation development is poor, with NT embryos showing relatively high levels of perinatal death and skeletal malformations [30], [55]. It is possible that the ethanol component of the activation protocol may also contribute to difficulties in obtaining live offspring. Iannaccone *et al* were able to successfully activate with strontium using a different culture medium than Hayes *et al*, although they were also unsuccessful at obtaining live animals [21].

These findings suggest that the somewhat crude methods of activation that have been used in other species are inadequate for rat nuclear transfer in general. DMAP is a general protein kinase inhibitor that induces activation by preventing the phosphorylation of cdc25, which is normally responsible for activating MPF [30]. However, DMAP is nonspecific, and will block numerous other kinase activities, leading to the abnormal mobilization of cytoskeletal components and premature mitosis [36]. Although the I+DMAP protocol worked reasonably well in the case of parthenogenic activation of oocytes, it mostly caused degeneration when used to activate reconstructed embryos. In general, we found that reconstructed embryos were more fragile than normal embryos or parthenotes, consistent with other published observations [20], [56], [57]. We substituted more specific CDKIs for DMAP in a similar protocol, and ultimately focused on bohemine. Unlike the other activation strategies attempted, ionomycin followed by bohemine resulted in similar rates of activation for both parthenogenic and reconstructed embryos. Other reversible CDKIs will likely be comparable to bohemine. It is possible that activation rates may be further improved through the use of alternative calcium ionophores, such as A23187 [58], [59], or by inhibitors of Ca^2+^-dependent ATPases (*e.g.*, thapsigargin [60]).

It has been shown that exposure to inorganic phosphate in the media induces a block at the 2-cell stage in the rat embryo [34], [61], [62]. To our knowledge, the best chemically defined medium reported for the culture of rat embryos is mR1ECM [34], a phosphate free media used in these studies. Rates of blastocyst development in this media were poor, <2% for both reconstructed embryos and normally fertilized rat oocytes. In contrast, ∼70% of fertilized mouse oocytes typically reach the blastocyst stage when cultured in KSOM [63]. It was recently reported that Sprague-Dawley (SD) oocytes will develop to the blastocyst stage in mR1ECM, but not those of four inbred rat strains [21]. However, this same study also reported a rate of blastocyst formation for reconstructed embryos of <10%. Although LEH rats are also an outbred strain, LEH derived embryos do not appear to fare as well *in vitro* as those derived from the SD strain. It is possible that a detailed, side-by-side comparison of LEH and SD oocytes may shed light on the mechanism involved. It is clear that mR1ECM is an inadequate media for the *in vitro* culture of reconstructed rat embryos in general. It is possible that some of these issues might be circumvented by transferring activated embryos to surrogate mothers no later than the 2–4 cell stage, or possibly immediately after exposure to activating conditions.

It may be possible to improve overall efficiency by performing additional modifications to culture conditions. The simplest alteration that has been successful in other systems has been the use of feeder cell layers, such as embryonic rat fibroblasts or buffalo rat liver (BRL) cells [64]. Feeder cells may release growth factors into the media or assist in the removal of toxic substances, and development rates can double or more in the presence of some form of helper cell. The addition of insulin [65], vitamins [66], or amino acids [63] might also be beneficial (insulin and amino acid supplementation alone can triple the rate of rat blastocyst development). Finally, serum is not a normal component of mR1ECM. Since serum is a source of lipids, minerals and hormones that are not present in normal media, the addition of a small amount of either fetal bovine serum or normal rat serum may dramatically improve *in vitro* development.

Numerous inefficiencies currently prevent the reproducible implementation of rat SCNT. In this study we improved substantially on existing methods of oocyte activation. However, activation efficiency is only a single facet of the problem. Inadequately definined culture conditions for rat embryos remains a central issue. This is a major obstacle to making this technology viable for rats, since rat embryos develop poorly *in vitro*. A better understanding of rat oocyte physiology, also essential for developing better models of disease, may also provide insights that will be useful for making the SCNT process more efficient. This approach may also be useful for embryos from other species that prove less amenable to *in vitro* manipulation.
